# Unusual Neuropsychiatric Presentation of *Cryptococcus neoformans* Meningoencephalitis in an Immunosuppressed Patient with Rheumatoid Arthritis: A Case Report

**DOI:** 10.3390/diseases13120404

**Published:** 2025-12-17

**Authors:** Sinthia Vidal-Cañas, Manuel David Mayoral-Valencia, Esteban Artunduaga-Cañas, Esteban Pineda-Arias, Danna Alejandra Betancourt Cañas, Daniela Arturo-Terranova

**Affiliations:** 1 Grupo de Investigación en Salud Integral (GISI), Facultad de Salud, Universidad Santiago de Cali, Cali 760035, Colombia; sinthia.vidal00@usc.edu.co; 2 Facultad de Salud, Universidad Tecnológica de Pereira (UTP), Pereira 660005, Colombia; manuel.mayoral@javerianacali.edu.co; 3 Facultad de Salud, Universidad del Valle, Cali 760035, Colombia; 4 Facultad de Salud, Pontificia Universidad Javeriana, Cali 760035, Colombia; 5 Facultad de Salud, Unidad Central del Valle del Cauca (UCEVA), Tulúa 763021, Colombia; esteban.artunduaga01@uceva.edu.co; 6 Grupo de Investigación en Genética, Fisiología y Metabolismo (GEFIME), Ciencias de la Salud, Universidad Santiago de Cali, Cali 760035, Colombia; 7 Semillero de Enfermedades Infecciosas Transmisibles (EINTRANS), Facultad de Salud, Unidad Central del Valle, Tuluá 763021, Colombia; pinedaariasesteban@gmail.com; 8 Facultad de Salud, Universidad Santiago de Cali, Cali 760035, Colombia; dannalejabetancourt@gmail.com

**Keywords:** case report, cryptococcosis, meningitis, cryptococcal, arthritis, rheumatoid, etanercept, delirium

## Abstract

Central nervous system (CNS) cryptococcosis caused by *Cryptococcus neoformans* is a severe opportunistic infection that primarily affects individuals with impaired cellular immunity. Although the classic presentation includes headache, fever, and meningeal signs, chronically immunosuppressed patients may develop atypical neuropsychiatric manifestations, leading to diagnostic delays. We report the case of a 53-year-old man with rheumatoid arthritis (RA) receiving long-term prednisolone and etanercept therapy, who presented with a 7-day history of depressive mood, anhedonia, social withdrawal, irritability, and progressive confusion. Neurological examination revealed disorientation without focal deficits. Brain imaging showed only mild cortical atrophy, and cerebrospinal fluid (CSF) analysis revealed lymphocytic pleocytosis, low glucose, and elevated protein levels. Multiplex PCR (FilmArray^®^) of CSF identified *Cryptococcus neoformans*, CSF positive to *C. neoformans*. The patient was treated with liposomal amphotericin B followed by fluconazole, resulting in gradual improvement of both neurological and psychiatric symptoms. This case highlights an unusual presentation of CNS cryptococcosis in a non-HIV immunosuppressed patient with RA, emphasizing that acute psychiatric or cognitive changes can be the predominant manifestation. Clinicians should consider fungal infections in the differential diagnosis of acute neuropsychiatric symptoms in patients receiving chronic corticosteroid and biologic therapy. Early recognition and molecular diagnosis can facilitate timely antifungal treatment, potentially improving prognosis and reducing morbidity associated with delayed therapy. This report underscores the importance of awareness of atypical presentations of opportunistic infections in immunosuppressed populations.

## 1. Introduction

Central nervous system (CNS) cryptococcosis, primarily caused by *Cryptococcus neoformans*, remains a severe opportunistic fungal infection characterized by high morbidity and mortality. Traditionally, this condition has been well documented among individuals with advanced HIV infection or solid organ transplantation, where profound cellular immunosuppression facilitates fungal invasion and dissemination [1]. However, over the last decade, an increasing number of reports have highlighted its occurrence in patients with autoimmune diseases, particularly rheumatoid arthritis (RA), who receive long-term immunosuppressive or biologic therapies [2,3]. This shift underscores the expanding clinical spectrum of CNS cryptococcosis beyond classical immunodeficiency syndromes, posing new diagnostic and therapeutic challenges in rheumatologic populations [4].

Rheumatoid arthritis is a chronic inflammatory disorder in which disease-modifying antirheumatic drugs (DMARDs), glucocorticoids, and targeted biologic or synthetic agents, such as tumor necrosis factor-alpha (TNF-α) inhibitors, Janus kinase (JAK) inhibitors, and interleukin-6 (IL-6) receptor antagonists, have improved disease control but have also modified infection risk profiles [5,6]. Corticosteroids remain a cornerstone of therapy; however, their chronic use contributes to cumulative immunosuppression. Similarly, biologic therapies such as etanercept or adalimumab, as well as JAK inhibitors like tofacitinib, baricitinib, and upadacitinib, have been associated with opportunistic fungal infections, including cryptococcosis [4,7].

Recent epidemiological studies and meta-analyses support the increased incidence of infections associated with these agents [8]. Ouranos et al. (2024) demonstrated a 1.7% cumulative incidence of opportunistic infections in RA patients receiving JAK inhibitors, with up to a 2.7-fold higher risk compared with placebo [3]. Yoshida et al. (2024) reported that patients treated with JAK or IL-6 inhibitors exhibited significantly higher infection rates than those receiving conventional DMARDs, particularly affecting the respiratory and central nervous systems [9]. Furthermore, Choi et al. (2023) compared JAK inhibitors with TNF inhibitors, observing that JAK inhibition conferred a modestly increased risk of serious infections, especially in older adults and patients with comorbidities [10]. Favalli et al. (2024) emphasized that while biologics and JAK inhibitors have distinct immunomodulatory mechanisms, both disrupt cytokine signaling critical to antifungal defense, particularly Th1-mediated immunity, predisposing patients to invasive fungal infections such as cryptococcosis [11]. Taylor (2023) highlighted that JAK inhibitors, despite their clinical efficacy in refractory RA, require continuous pharmacovigilance due to interference with interferon-mediated host defense mechanisms [12]. Together, these findings underscore the need for heightened clinical suspicion when RA patients on these therapies present with atypical neurological or psychiatric symptoms.

Although the classical triad of meningitis—fever, headache, and neck stiffness—remains well recognized, CNS cryptococcosis in chronically immunosuppressed patients may present with subtle or atypical features such as cognitive decline, depression, anhedonia, social withdrawal, or behavioral changes, often delaying diagnosis and treatment initiation [1,2]. The absence of overt meningeal signs challenges conventional diagnostic pathways, highlighting the importance of integrating neuroimaging, cerebrospinal fluid (CSF) molecular testing, and fungal antigen detection in the early evaluation of neuropsychiatric syndromes among immunocompromised patients [13,14].

We describe a case of cryptococcal meningoencephalitis due to *C. neoformans* in a 53-year-old man with RA treated with prednisolone and etanercept, who presented primarily with acute neuropsychiatric disturbances in the absence of meningeal signs. The multiplex PCR panel (FilmArray^®^) allowed rapid and definitive identification of *C. neoformans*, which enabled the immediate initiation of targeted antifungal therapy. This molecular diagnosis was later corroborated by CSF culture, which became positive on day 27. This case underscores the utility of rapid molecular diagnostics and emphasizes the need to include fungal etiologies in the differential diagnosis of acute neuropsychiatric disorders in patients undergoing long-term immunosuppressive therapy for RA.

## 2. Case Report

**Figure 1:**
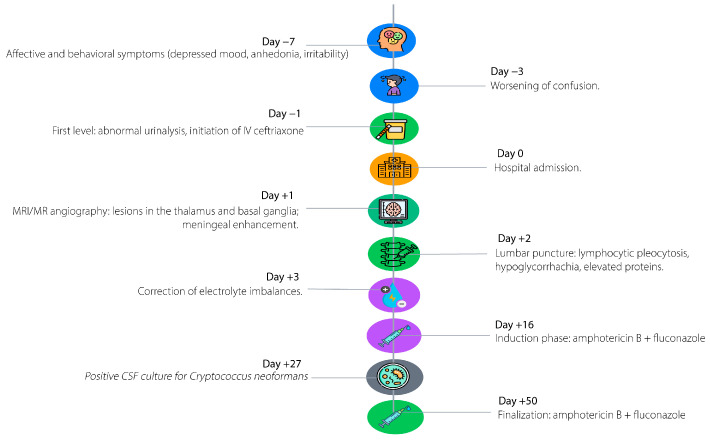
Timeline of clinical events.

Figure 1 illustrates the key chronological milestones in the patient’s clinical course, starting seven days prior to admission with progressive behavioral and affective changes, including depressive symptoms, irritability, anhedonia, social withdrawal, and anorexia. Three days before admission, the patient developed rapidly worsening neurological deterioration, characterized by confusion, psychomotor slowing, mild dysarthria, reduced mobility, vomiting, and urinary incontinence, prompting evaluation at a primary care facility and empiric treatment with intravenous ceftriaxone for presumed urinary tract infection with delirium. On the day of hospital admission, advanced neuroimaging revealed focal hyperintense lesions in the right thalamus and globus pallidus. Forty-eight hours post-admission, cerebrospinal fluid analysis demonstrated lymphocytic pleocytosis, hyperproteinorrhachia, and hypoglycorrhachia. Three days later, multiplex PCR confirmed *Cryptococcus neoformans*, guiding the prompt initiation of targeted antifungal therapy with liposomal amphotericin B and intravenous fluconazole. Subsequent hospitalization included correction of electrolyte disturbances and progressive clinical improvement in consciousness and verbal fluency.

### 2.1. Initial Presentation and Medical History

We present a 53-year-old urban male resident with a long-standing history of rheumatoid arthritis. The patient had been on chronic treatment with prednisolone and etanercept for over two years, leading to prolonged immunosuppression. He also had exogenous Cushing syndrome secondary to corticosteroid use, recurrent abdominal furunculosis, and grade I obesity. He reported no history of alcohol, tobacco, or illicit drug use, and no previously documented opportunistic infections.

The clinical course began seven days prior to admission with progressive behavioral and affective changes, including depressive symptoms, easy crying, irritability, anhedonia, social withdrawal, and marked anorexia. During the three days before transfer, he developed rapidly worsening neurological deterioration, including a confusional state, psychomotor slowing, mild dysarthria, global reduction in mobility, vomiting, and urinary incontinence, prompting consultation at a primary care facility. An abnormal urinalysis led to initiation of intravenous ceftriaxone under the presumption of a urinary tract infection with associated delirium. However, persistence of neurological impairment prompted referral to the presenting institution.

### 2.2. Clinical Examination and Laboratory Evaluation

On admission, the patient was hemodynamically stable, with a blood pressure of 130/80 mmHg, heart rate of 75 bpm, respiratory rate of 16 breaths per minute, temperature of 36.2 °C, and oxygen saturation of 94% on room air. General physical examination revealed normocephaly, anicteric sclera, moist oral mucosa, symmetric and normally expansible chest, regular heart sounds without murmurs, and a soft, depressible abdomen without masses or organomegaly. Neurological evaluation showed disorientation in all three spheres, bradypsychia, and hypofluent speech with mild prosody impairment, with no focal motor or sensory deficits and no meningeal signs observed.

**Figure 2:**
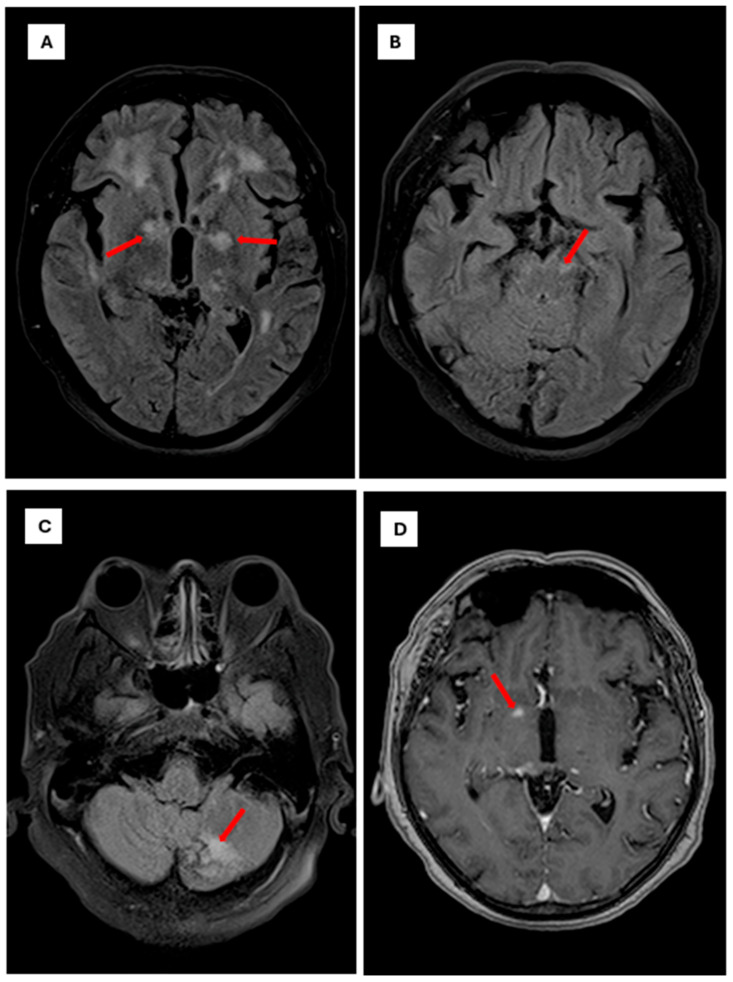
Brain magnetic resonance imaging findings in Cryptococci meningoencephalitis. Note: Axial brain MRI sequences demonstrating characteristic findings of cryptococcal meningoencephalitis The scale bar is set to 10 mm. (**A**): Axial FLAIR sequence showing focal hyperintense lesions involving the white matter and bilateral gangliobasal region, particularly the globus pallidus (red arrows). (**B**): Axial FLAIR sequence showing a focal hyperintense lesion affecting the left mesencephalic peduncle (red arrows). (**C**): Axial FLAIR sequence showing a focal hyperintense lesion involving the posterior lobe of the left cerebellar hemisphere (red arrow). (**D**): Axial T1-weighted sequence with gadolinium contrast showing a pinpoint focal enhancement in the right globus pallidus (red arrow).

**Figure 3:**
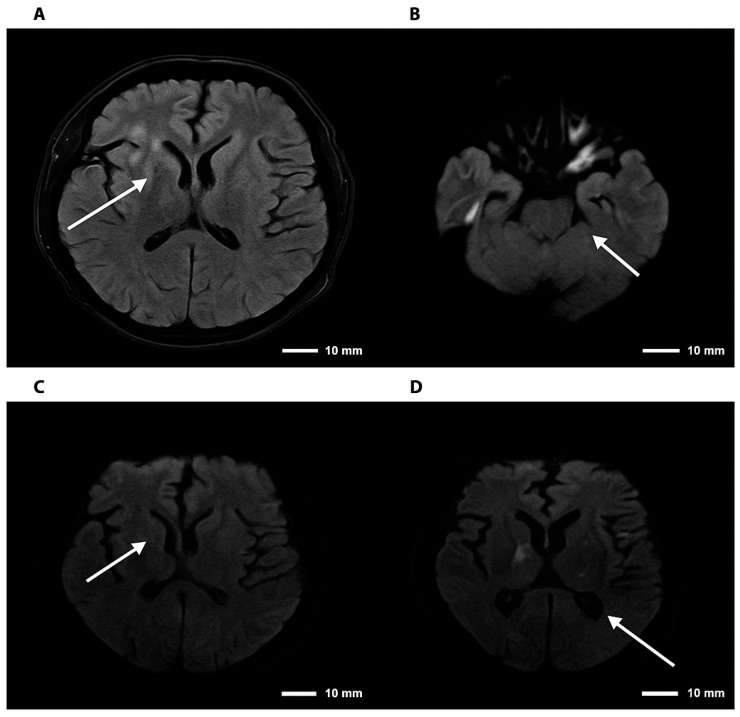
Brain magnetic resonance imaging—FLAIR and DWI sequences in cryptococcal meningoencephalitis. Note. Brain magnetic resonance imaging. (**A**) Axial FLAIR sequence showing hyperintensity in the right basal ganglia. (**B**) DWI sequence demonstrating diffusion restriction in the right thalamic region. (**C**,**D**) Axial DWI images confirming foci of restriction in the right basal ganglia and thalamus, consistent with an infectious/inflammatory process. The arrows indicate lesions consistent with cryptococcomas, involving the supra- and infratentorial parenchyma to varying degrees.

Initial laboratory studies revealed a blood glucose level of 78 mg/dL, C-reactive pro-tein of 1.7 mg/L, sodium of 128 mmol/L, and hyperkalemia of 5.4 mmol/L, with normal renal and hepatic function. Glycated hemoglobin was 5.3%. Given the patient’s immuno-suppression and rapid neurological progression, advanced neuroimaging was performed. Brain MR angiography demonstrated mild segmental stenosis in the middle and anterior cerebral arteries without significant occlusions; complete cerebral angiography was nor-mal. Brain MRI revealed focal hyper intense lesions in the right thalamus (10 × 6 mm^2^) and Globus pallidus, suggestive of infectious or inflammatory etiology (Figure 2) and (Figure 3).

Lumbar puncture revealed clear cerebrospinal fluid (CSF) with elevated protein (366–500 mg/dL), reduced glucose (26–32 mg/dL; serum glucose 133 mg/dL), and lymphocytic pleocytosis (100–217 cells/µL, 96% lymphocytes). Additional CSF parameters included LDH 82.3 U/L, albumin 1.0 g/dL, pH 7.5, and specific gravity 1.015, with >1000 RBCs/µL attributable to a traumatic tap. The initial diagnosis was established by the detection of *C. neoformans* via PCR (FilmArray^®^), which provided definitive evidence for the causative pathogen and enabled prompt therapy; this finding was subsequently confirmed by a positive CSF culture reported on day 27 (Table 1).

After multiplex PCR detected *Cryptococcus* spp., cerebrospinal fluid was cultured on Sabouraud agar with dextrose (AV-LAB), a selective medium commonly used for yeast isolation, and simultaneously on blood agar to improve recovery of the organism in low-load infections. Since *Cryptococcus* species grow slowly, both media were incubated for 10 days under standard mycological conditions. Once the yeast colonies were obtained, species-level identification was performed using the VITEK® automated microbial identification system (bioMérieux, Marcy-l’Étoile, France). which evaluates carbohydrate assimilation and enzyme profiles with validated accuracy to distinguish *C. neoformans* from other pathogenic yeasts. The biochemical pattern generated by VITEK^®^ was compatible with *Cryptococcus neoformans*, providing definitive confirmation of the etiological agent.

### 2.3. Hospital Course and Management

The diagnosis of cryptococcal meningoencephalitis was confirmed in an immunocompromised patient receiving chronic corticosteroid and anti-TNF therapy. Induction therapy was initiated with liposomal amphotericin B (3 mg/kg/day, 250 mg intravenously every 24 h) and intravenous fluconazole (400 mg/day). Although combination therapy with liposomal amphotericin B and flucytosine is the recommended induction standard, flucytosine was unavailable at our institution and therefore was not included in the initial regimen. Premedication included oral acetaminophen (1 g) and loratadine (10 mg), and prehydration with 1000 mL of Hartmann’s solution over 2 h was performed to reduce nephrotoxicity.

During hospitalization, the patient developed hypokalemia (nadir 3.0 mmol/L) and mild hyponatremia, both of which were successfully corrected with oral and intravenous supplementation. Renal function remained stable (creatinine 0.8 mg/dL; BUN 10.9 mg/dL). Clinically, the patient showed progressive improvement in consciousness and verbal fluency after ten days of therapy, although mild psychomotor slowing persisted. Follow-up MRI was planned to monitor the evolution of cerebral lesions and guide the transition to the consolidation phase of antifungal therapy.

## 3. Discussion

### 3.1. Main Findings and Comparative Analysis

Cryptococcal Cryptococcal meningoencephalitis remains a serious CNS infection with high morbidity and mortality, even in the era of modern antifungal therapies [1]. While extensively characterized in patients with HIV or transplant recipients, cases associated with autoimmune diseases under immunosuppressive therapy have increasingly been reported over the past decade [1,2]. These cases often present atypically, delaying diagnosis and treatment.

In rheumatoid arthritis (RA), the risk of opportunistic infections is heightened by prolonged use of corticosteroids and by targeted immunosuppressive therapies, including biologic agents and JAK inhibitors [4,5]. Liao et al. (2016) demonstrated that, among patients with rheumatoid arthritis, chronic kidney disease and exposure to the monoclonal anti-TNF antibody adalimumab were significantly associated with an increased risk of cryptococcal infection [4]. Similarly, our patient received combination therapy with prednisolone and etanercept, both potent modulators of cellular immunity.

Structured reviews and case reports support these observations. Winthrop et al. (2023) reported that treatment with JAK inhibitors in rheumatoid arthritis is associated with an increased risk of opportunistic infections —including fungal infections— particularly in patients with prolonged exposure, advanced age, concomitant immunosuppression, or multiple comorbidities [5]. Individual reports describe atypical presentations, with nonspecific cognitive and behavioral changes and absence of classic meningeal signs, as seen in our patient [6,7,8,12,13]. Moreover, TNF inhibitors and other biologics may predispose to CNS infections by impairing cellular immune responses and possibly disrupting the blood–brain barrier [8,9]. Recent studies indicate that TNF inhibitors increase the incidence of invasive fungal infections [8], and that JAK inhibitors may confer an even broader risk due to their cytokine-signaling inhibition [3,5].

Neuroimaging findings in CNS cryptococcosis are variable, with lesions mimicking tumors, ischemic events, or demyelination, emphasizing the need for integrating advanced imaging with molecular diagnostics for early and accurate detection [13,14].

Neuropsychiatric manifestations can predominate, with involvement of subcortical and limbic structures leading to behavioral and cognitive changes rather than classical meningitis symptoms, consistent with cases reported in both immunocompetent and immunosuppressed patients [15,16,17,18].

Further imaging-based studies have described cryptococcal lesions with heterogeneous radiological patterns, including those reported in recent systematic reviews, reinforcing the complexity of CNS involvement [19].

Despite the CSF revealing lymphocytic pleocytosis and hypoglycorrhachia compatible with infectious meningoencephalitis, it is essential to highlight that the India ink stain was negative and the initial CSF culture was negative until day 27, a finding previously documented in immunosuppressed patients with cryptococcosis [20]. This low sensitivity of the India ink in our case, along with the initial negative culture, contrasts sharply with the early and definitive detection of C. neoformans using multiplex PCR (FilmArray®). This discrepancy underscores the high diagnostic sensitivity of molecular methods, making them crucial in scenarios involving a low fungal burden.

The fact that the infection was predominantly localized in the cerebral parenchyma—evidenced by the focal hyperintense lesions in the thalamus and globus pallidus on MRI—and not in the subarachnoid space, may have resulted in a low concentration of antigen or fungal cells in the CSF. This localization in subcortical and limbic structures explains why atypical neuropsychiatric and cognitive manifestations predominated over classical meningeal signs, reinforcing the need to incorporate PCR into the diagnostic evaluation of acute neuropsychiatric syndromes in immunosuppressed patients.

To contextualize these findings, Table 2 summarizes representative cases reported in the literature, together with other published evidence not individually detailed here.

### 3.2. Clinical Implications

This case highlights that CNS cryptococcosis may present primarily with neuropsychiatric symptoms in RA patients receiving immunosuppressive therapy, necessitating high clinical suspicion even in the absence of fever, headache, or nuchal rigidity. Early initiation of antifungal therapy—preferably liposomal amphotericin B with flucytosine followed by fluconazole maintenance—is essential to improve outcomes [1,2].

Temporary discontinuation of biologic therapy may be considered during antifungal treatment to support immune recovery, balanced against the risk of RA flare [4,19,20]. Notably, IL-6 blockade with tocilizumab may offer a relatively safer infection profile compared with TNF or JAK inhibitors [2,19].

Preventive strategies should include risk stratification before initiating biologics or JAK inhibitors, as well as patient education to recognize early signs of infection. Screening for latent fungal infections may be considered in high-risk patients receiving prolonged or combined immunosuppression [3,5,19].

### 3.3. Limitations

This report is limited by its single-patient design, which constrains generalizability. Imaging and molecular diagnostic findings were specific to this case, and broader epidemiological conclusions cannot be drawn. Furthermore, long-term outcomes and neuropsychiatric sequelae may vary among patients, and the contribution of chronic inflammation versus direct fungal injury to cognitive impairment remains unclear.

### 3.4. Future Recommendations

Further research is warranted to better characterize the clinical manifestations and outcomes of cryptococcal infections in patients with autoimmune diseases receiving combined immunosuppressive therapy, particularly corticosteroids and anti-TNF agents. Larger case series and multicenter studies are needed to define early diagnostic markers, optimal antifungal regimens, and appropriate treatment durations in this specific population.

We recommend implementing routine cryptococcal antigen screening in high-risk patients with rheumatoid arthritis or other autoimmune conditions undergoing prolonged or combined immunosuppressive therapy, especially before initiating biologic agents. Additionally, clinicians should maintain a high index of suspicion for central nervous system involvement when these patients present with headache, confusion, or subtle neurocognitive changes.

Enhanced awareness, early diagnostic testing, and standardized management protocols could significantly improve outcomes and reduce morbidity associated with opportunistic fungal infections in immunosuppressed individuals.

## 4. Conclusions

Cryptococcal meningoencephalitis in patients with rheumatoid arthritis receiving biologic therapies represents a major diagnostic and therapeutic challenge, particularly when it presents with atypical neuropsychiatric manifestations and absence of classical meningeal signs. The evidence reviewed indicates that the risk of this opportunistic infection is strongly influenced by the combination of immunosuppressive agents, duration of therapy, and underlying comorbidities, rather than by a single pharmacological agent.

In this context, early identification requires a high index of clinical suspicion, especially in the setting of acute neuropsychiatric syndromes in immunosuppressed patients. The integration of advanced neuroimaging with molecular diagnostic techniques in cerebrospinal fluid can shorten the time to antifungal therapy initiation, which is crucial to reducing morbidity, mortality, and neurological sequelae.

This case broadens the clinical spectrum documented in the literature and reinforces the need to include CNS cryptococcosis in the differential diagnosis of acute-onset cognitive and behavioral disorders in this patient population. Furthermore, it underscores the importance of personalized clinical surveillance strategies and multidisciplinary collaboration among rheumatology, infectious diseases, and neurology to optimize outcomes.

## Figures and Tables

**Table 1:** Relevant paraclinical results for the diagnosis and follow-up of *Cryptococcus neoformans* meningoencephalitis in an immunosuppressed patient with rheumatoid arthritis.

Parameter	Result	Units	Clinical Relevance
Serum glucose	78	mg/dL	Baseline value for CSF glucose interpretation
C-reactive protein (CRP)	1.7	mg/L	Low systemic inflammatory response
Serum sodium	128	mmol/L	Hyponatremia associated with CNS infection
Initial serum potassium	5.4	mmol/L	Initial hyperkalemia
Serum potassium (follow-up)	3.0	mmol/L	Hypokalemia secondary to treatment
Creatinine	0.8	mg/dL	Normal baseline renal function
BUN	10.9	mg/dL	Preserved renal function
CSF FilmArray^®^	*Cryptococcus neoformans*	—	Etiological confirmation
CSF—Protein	366–500	mg/dL	Marked hyperproteinorrhachia
CSF—Glucose	26–32	mg/dL	Significant hypoglycorrhachia
Simultaneous serum glucose	133	mg/dL	CSF/serum ratio < 0.3
CSF—White cells	100–217	cells/µL	Lymphocytic pleocytosis (96% lymphocytes)
CSF—LDH	82.3	U/L	Elevation consistent with fungal infection
CSF—Albumin	1.0	g/dL	Blood–brain barrier disruption
India ink stain	Negative	—	Limited sensitivity in immunocompromised patients
CSF culture TBC	Negative	—	--
Brain MRI	Focal lesions in right thalamus (10 × 6 mm^2^) and globus pallidus	—	Findings compatible with cryptococcal meningoencephalitis
Cerebral panangiography	No abnormalities	—	Ruled out significant vasculitis

Abbreviations: CRP, C-reactive protein; BUN, blood urea nitrogen; CSF, cerebrospinal fluid; LDH, lactate dehydrogenase; MRI, magnetic resonance imaging.

**Table 2:** Comparison of relevant articles.

Author	Immunosuppressive Therapy	Clinical Presentation	Diagnosis
Liao et al., 2016 [4]	Adalimumab ± corticosteroids	Fever, headache, confusion, meningeal signs in some cases	CSF culture, imaging
Winthrop et al., 2023 [5]	JAK inhibitors	Opportunistic infections with variable clinical features	Blood or tissue culture, PCR, antigen testing
Prakash et al., 2020 [6]	Ruxolitinib	Nonspecific cognitive symptoms, mild headache, delayed meningeal signs	CSF culture
Cases et al., 2022 [7]	Ixekizumab (IL-17 inhibitor)	Confusion, headache, focal neurological deficits	CSF culture, MRI
Li et al., 2020 [8]	Anti-TNF agents (infliximab, adalimumab), etanercept	Fever, headache, altered mental status	CSF culture
Yoshida et al., 2024 [9]	JAK or IL-6 inhibitors	Acute confusion, disorientation, psychomotor agitation, mild fever	CSF culture, PCR
Choi et al., 2023 [10]	JAK vs. TNF inhibitors	Cognitive impairment, drowsiness, behavioral changes	CSF analysis, MRI

Abbreviations: CSF, cerebrospinal fluid; MRI, magnetic resonance imaging; PCR, polymerase chain reaction; TNF, tumor necrosis factor; IL, interleukin; JAK, Janus kinase.

## Data Availability

Data contained within the article.

## Abbreviations

The following abbreviations are used in this manuscript:

RA	Rheumatoid arthritis
TNF-α	tumor necrosis factor-alpha
BUN	Blood Urea Nitrogen
CNS	Central Nervous System
CRP	C-Reactive Protein
CSF	Cerebrospinal Fluid
DWI	Diffusion-Weighted Imaging
DMARDs	Disease-Modifying Antirheumatic Drugs
FLAIR	Fluid-Attenuated Inversion Recovery
IL-6	Interleukin-6
